# Mr. O'Halloran on the Yellow Fever of Andalusia, during the Epidemic of 1820

**Published:** 1822-03-01

**Authors:** 


*A brief View of the Yellow Fever, as it appeared in Andalusia, during the Epidemic of* 1820: *together with the Mode of Treatment adopted, and an Account of the Appearances on Dissection. To which is prefixed, a short Topographical Sketch of the Country.* By Thomas O'Halloran, Esq. Octavo, pp. 171. London, 1821.

The dreadful epidemic of which this work treats, has, as usual, suspended its ravages till the elements are again disposed to reproduce it—or, until ignorance, prejudice, or crooked policy, shall invent a new tale of its importation from a distant soil. The best historical records inform us, that the first great sickness that nearly depopulated Cadiz, appeared in 1466. A similar event took place in 1507. The epidemic of 1582 is *said* to have ceased through the intercession of St. Roque! It was not, however, until the year 1730, that the disorder, since known by the name of "el vomito negro," or black vomit, first made its appearance, and swept off great numbers of the inhabitants. In the year following it was equally dreadful, exhibiting spots of a livid yellow, or dark colour, that covered the body, and were the certain forerunners of the black vomit. Don Josef Cervi, physician to Charles the Third, declared that it was *not* the plague; and Don F. Navarette affirmed that this disease (el vomito negro) was introduced into Cadiz, by *a vessel from Spanish America* —a tale that has been repeated by one *importer* after another, down to the present day, when it has become, not only flat, stale, and unprofitable, but also, exceedingly pernicious.

In 1764, a similar disorder appeared in Cadiz, which was witnessed by our immortal countryman, Dr. Lind, who has written an account of the disease. Dr. Salvaresa, a famous Spanish physician, has also written a Latin account of the same epidemic. Both accounts exactly agree in describing the disease, precisely as it has appeared in the late epidemic; but Salvaresa says nothing about its being contagious—on the contrary, he attributes it to atmospheric causes and corrupted corn, while, Lind so wavers in his opinion, that nothing decisive can be gleaned from it.

During a period of sixty years after the last-mentioned fever, Cadiz remained healthy, notwithstanding the progressive increase of population. The last months of 1799, and beginning of 1800, were characterized by remarkably severe weather, so that, at the end of May, there was scarcely any appearance of Spring. All at once, however, "the heat of Summer (we quote Sir James Fellowes, a decided contagionist and *importer)* set in from the *beginning* of June, and by the month of August, the mercury was 90° Fahrenheit, while the *Levanter* tended to increase the distress, which the intense heat of the weather generally occasioned."*

During the months of June and July, no material alteration took place in the public health; but in the beginning of August, the scene began to change. A fever broke out in the district of St. Maria, (the residence of poverty, the theatre of filth, where are situated the posadas, taverns, and lodging houses, for the dregs of society) whence it radiated in all directions, and in whatever house it appeared, all the family was ultimately attacked. About the middle of August, all heads were laid together to discover the cause and origin of the epidemic, and, as in all such cases, the Junto pitched upon a ship, the Dolphin, from Spanish America, on board of which ship, some smugglers from the shore had been, and must, of necessity, have brought the infection into the town.

By the middle of September the diurnal mortality amounted to 200, and Cadiz presented a most melancholy scene of mourning and desolation. At this time, according to the statements of the contagionists and importers themselves, and among others Sir James Fellowes, the disease *imported from Spanish America* spread to domestic and other animals, and *dogs and cats* were seen dying with the *black vomit.* The very horses died. This is a fair specimen of the credulity of the *importers*—whose creed indeed appears generally to be —"credo *quia* impossibile." But the worst of it is, that credulity is almost always accompanied by an illiberal and overbearing disposition, which endeavours to stifle fair and rational investigations of truth, by its imperious ipse dixits. We every day see examples of this, both at home and abroad, but we shall here notice a remarkable instance in point. Mr. Doughty, a zealous and intelligent surgeon, who had seen and suffered from yellow fever in the West Indies, and who acted under Sir James Fellowes at Cadiz, in 1810, readily recognized a striking similarity, if not an identity, between it and the fever of Cadiz. This officer having opened some bodies and found the most decisive marks of inflammation in some of the internal organs, reported the same to Sir James Fellows, but that cautious physician never acknowledged the receipt of the letter, nor took any notice of it in his publication. Nay, because Mr. Doughty stated in a letter to the Duke of Kent that he was the only officer who had re-

* Reports, p. 33.

course to dissection, he was brought to a court martial by Sir James, and dismissed the service. Mr. Doughty states, "that he never had the honour of seeing Sir James Fellowes by the side of any one of the many bodies he opened." No, no. The contagion importers act on a very different principle. Like Falstaff, they are mighty shy in the engagement, but once they get to a distance from the scene of action, we are deafened with the narratives of what they have seen and done.

It would be endless to notice the absurd tales invented by *importers* of contagion. Thus when, in 1810, the fever raged at Barcelona, Dr. Risneno, Physician to the Spanish Hospital there, positively asserted that the contagion was brought *from* Gibraltar and Cadiz; while Dr. Pym as positively asserts that it was brought *to* Gibraltar from Carthagena!

Our readers are aware that the doctrine uniformly advocated in this Journal, and we believe very generally admitted in this country, is that of *contingent* contagion in fever. We are not so mad, or so blinded by prejudice, as to believe with the ultra or *importing* contagionists, that the epidemics which have, from time to time, ravaged Spain, America, the coasts of the Mediterranean, and other parts of the world, were never of local origin in those places, but always transmitted by bales of goods or other vehicles from distant sources; nor can we agree with the anti-contagionists that such fevers, when once produced by aerial or terrestrial influence, are totally incapable of communication from individual to individual afterwards. But we do most solemnly protest against that barbarous doctrine which would barricade the sick, like so many rabid animals, and leave them to *form* an increasing focus of that contagion which it ought to be the duty of the physician and legislator to dilute, dissipate, and prevent. We consider plague as a distinct disease, *sui generis,* and do not object to its circumvallation. But to throw cordons of troops round those places where the *causes* of the fever are perpetually extricating themselves from the soil, at particular seasons, and thus prevent the wretched inhabitants from leaving the insalutary city or district, is one of the greatest cruelties that purblind man was ever permitted, by Providence, to inflict on his fellow creatures. It is a measure disgraceful to medical science, because it is contrary to the best established principles upon which we act—it is disgraceful to human nature, because it violates and uproots all feelings of charity and philanthropy towards those who are most entitled to our commiseration, and most in need of our assistance. In every quarter of the globe medical evidence is fifty to one in favour of separating the sick, and removing them out of the sphere both of the febrific causes that first produced them, and the accumulated effluvia emanating from their own bodies afterwards. The records most worthy of credit prove satisfactorily that the above measures may be carried into effect, with perfect security to those residing in the vicinity of places affected with endemic and epidemic fevers. On the western side of the Atlantic this scarcely admits of a doubt, even among the contagionists of those countries; and as the *importers* trace the fevers of Spain to the new world, they must admit them to be governed by the same laws on one as on the other side of the Atlantic. As for expecting any thing like scientific information or independence of opinion from the Spanish practitioners themselves, it is quite useless. The degraded state of medicine in Spain is ably and forcibly drawn by Don Matéos, of Madrid, in his "Philosophy of Legislation," from which we shall make a few extracts for the edification of those few in this country who place implicit faith in the contagion creed of their Spanish brethren.

"Why then does medicine continue among the Spaniards in a state of infancy, bordering on barbarism? It is because the most noble and useful of professions is there regarded as a vile trade or contemptible occupation! It is because medical students, reduced by turns to mendicity or servitude, are classed with the apprentices of masons and shoe-makers! That man must be imbued with a more than ordinary philanthropy, who could dedicate genius or talent to a profession so unjustly, so unwisely dishonoured."

"The importance of public hygiene is generally acknowledged; but its utility is not sufficiently appreciated. Governments have not duly estimated the value of medical philosophers; and it is to a neglect of their council, that our prisons, barracks, and hospitals have become, in turns, the most frightful depôts of pestilence, and too often the graves of their wretched inhabitants! The practice of interment in churches is an abomination; it is to burn an incense on the alters of the Almighty, the odour of which may spread infection like pestilential miasmata!"

Don Matéos ranks himself among the anti-contagionists, and asserts that the dreadful fevers which devastated Madrid in 1803-4, presented the characters of inflammation—were rarely of a putrid nature—and required the constant operation of antiphlogistics, particularly blood-letting, which was of singular efficacy. The picture which this enlightened Spaniard draws of a public hospital is frightfully melancholy to contemplate.

"Figure to yourself," says he, "a vast edifice, whose architecture is calculated to astonish by its majesty, or please by its elegance, but whose construction is devoid of every thing that can contribute to salubrity! The medical officers are never consulted in any part of the fabrication or internal œconomy of the institution. Under the appellation of HEALERS (guérisseurs) the physicians, badly paid, and little respected, sink into insignificance, and become as careless and negligent as the host of menial attendants. The miserable patient, who hopes for succour and consolation from his doctor, can scarcely address him when he is passed by without an answer! Charged with the office of visiting a great number of sick, the physician sees little or no sickness. The food and the medicines most slovenly prepared, are still more negligently distributed. The linen, and other furniture of the kind, are bad in quality, and not regularly changed. The atmosphere of the wards is impregnated with fetid exhalations and no means of ventilation or purification are put in force to remedy the evil. In vain does the unhappy patient look around for some sensible being who may commiserate his sufferings —wherever he turns his eyes, the most doleful objects are presented to his view. Abandoned by their parents or friends, overwhelmed with chagrin and sickness, some are poisoned by the blunders of the stupid apothecary, while others, fall victims to the ignorance of the surgeon, who having made three or four unsuccessful attempts on the vein, in the fifth goes dash into the artery!"

Don Matéos concludes his work by deploring the profound state of abjection into which the healing art is plunged in Spain.

"Will it be believed," says he, "that in the Universities the Professors of Divinity, of Law; nay, even the Tutors themselves, would blush to be seen in company with the Professors of Medicine! The most petty artizan would be despised by his fraternity if he brought up his son as a Physician! The Government promotes, with all its influence, this strange perversion of ideas, this worse than Vandalism! It heaps humiliations on the heads of those benefactors of mankind; and seems to fear lest the learned Physician and Philanthropist should unmask sacrilegious abuses, and lay open those crimes which, though *authorized*, are not the less detestable."

After reading these extracts, will professional men on this side of the Channel expect to reap any other information from the health officers of Andalusia than what may happen to suit the views of their Government? No, indeed! The doctrine of importation will, of course, be the doctrine of Government, and it will be that vociferated by the health-officers in its employ.

We now come to Mr. O'Halloran's work. Our readers are aware that in our last number we gave an account of Dr. Jackson's essay on the same epidemic,* wherein was mentioned the zeal and intelligence of Mr. O'Halloran, who ac-

---

* One of our cotemporaries has hinted that we have not given quite a companied our venerable countryman through this arduous and dangerous undertaking.*

Mr. O'Halloran's work is divided into seven chapters, and an appendix. The first chapter presents an interesting topographical sketch of Andalusia, from which we shall extract a few particulars. The River *Tinto*, deriving its name from the yellow colour of its waters, rises in the Sierra Morena mountains, and has the property of petrifying and hardening sand, in less than a year, and forming into large masses those stones which happen to come into contact with one another. No verdure is seen on its banks—no fish can live in its stream. An immense plain, extending sixteen or seventeen miles along the banks of the Guadalete, and approximating the town of Xeres, near the Carthusian Convent, "*is usually inundated, during the heavy rains of winter and of spring, and is not entirely dry even in the hottest weather.*" We beg to draw the attention of our brethren to this fact, for every one who knows any thing of medical topography, must be convinced, that such a situation is peculiarly well calculated to produce fever, whenever the state of the air is favorable for the extrication of those febrific agents, that are known to have their fons et origo in such localities. At the extremity of the above-mentioned flat, near St. Lucar, a river takes its rise, and, after a circuitous route, empties itself into the Guadalete, close to the Carthusian convent. "*The bed of this river is partly dry in summer.*"

"It is to the country watered by this river, which for the most part is inundated during the winter months, that the ancients are supposed by some, to have given the name of the Elysian Fields; while the river which runs nearly at a right angle with the former, was called the GUADALETE or river of *Oblivion*. It is not, perhaps, surprising that the country in the *vicinity* of those rivers should have been denominated the "fields of bliss;" but how a muddy river, bounded on both sides by swamps of considerable extent, from PUERTO SANTA MARIA to the mountains near ARCOS, and without a single tree to embellish its banks, could have been taken for the celebrated river LETHE, I am at a loss to conceive." 6.

The town of Xeres itself, stands somewhat elevated at the extremity of this plain, commanding an extensive view of those elysian fields. "It is nearly surrounded, during the

---

fair representation of Dr. Jackson's work or sentiments; but we appeal to the author himself, and to the public at large, for the fidelity of our analysis on that, and every other occasion, without the slightest fear of being found wanting on the side of either correctness or candour.—*Rev.*

* See page 487, vol. ii.

prevalence of heavy rains, by swamps, the smallest of which is close to the town, and the greatest, at a distance of about three miles." Intermittent and remittent fevers, *annually* attack the inhabitants in great numbers, being "exceedingly difficult of management, and not unfrequently fatal in their termination." P. 8. Who but an *importer* would be blind to such obvious sources of disease, or obstinately insist, that contagion was annually smuggled in here, at one particular season of the year, but never at any other season?

Xeres is three miles in circumference, and contains 40,000 souls. It has a few broad, and tolerably well-paved streets, and some high and well-ventilated houses, in the centre of the town.

" But in other parts, with the exception of two small squares, the houses are ill constructed, the streets narrow, and either badly paved or not at all. But the worst is, that the streets in general, throughout their whole length, are covered in the centre with filth and ordure of all hues, collected from every house, so that passengers have only a narrow foot-way upon which they can walk with cleanliness or safety. Pools of black, stinking, stagnant water appear on the different streets, with few exceptions; and the intermediate spaces are covered with solid filth, which, when disturbed by the wheel of a carriage, or any other accidental occurrence, emits a stench that is absolutely intolerable to persons unaccustomed to it, not merely disgusting the sense, but, in many instances, actually turning the stomach. This loathsome condition of the town is, of course, ascribable to scarcity of sewers or drains to carry off the impurities which are daily and hourly thrown into the streets, where they are allowed to remain a perpetual memorial of the filthiness of the inhabitants. Some of the streets in the suburbs, where the poorer classes dwell, are dirty beyond description: a horse will sink to the knees in one of them, while the foot-passenger makes a perilous progress by pathways so narrow as scarcely to permit two abreast. The houses are low, crowded, ill ventilated, and commonly dirty within. The fronts are usually long; and a door in the centre serves for the entrance into the house, and to a square yard, or court, which is generally formed in the rear, by the junction of four different dwellings, the abodes of several families. The houses here being, in general, but one story high, become insufferable from the direct application of a burning sun during the summer and autumnal months. The tiles with which the roofs are covered, are heated to such a degree, as to make the inside a perfect oven, not inhabitable by persons unaccustomed to such exhausting and sickening heat. It often happens in these squares, that every room is the abode of a separate family; a solitary door giving ventilation and light to one or more apartments in which the several miserable objects are crowded together, with scarcely any accommodation except the mantas or rags which they spread on the bare ground, and upon which they lie. Those ill of fever, or other disease, are placed in the corner of the room, whilst he rest occupy the other parts of the same chamber, for days and nights, without the slightest repugnance or apprehension of danger." P. 11.

Let the *importers* of contagion peruse the above extract, and let every rational and unbiassed man, in this country, judge of the insanity, not to say inhumanity, of that horrible doctrine, that would circumvallate human beings, afflicted with endemic or epidemic diseases, in such pestiferous abodes!

"It is under circumstances, such as I now describe," says our author, "where ten or fifteen persons occupy the same ill-ventilated room, that disease commits its most terrible ravages; few, in such cases, escaping with life, when seriously attacked." 12.

Seeing then, that humanity is deeply interested in counteracting that detestable doctrine of importation, which entails such misery on thousands and tens of thousands of innocent people, we deem it our duty, to give all possible circulation to those *facts* collected by our own countrymen, on which we can better depend, than on the interested and distorted *reports* of foreigners. On this account, we entreat the patient attention of our readers, and the most deliberate consideration of all those, who feel for the afflictions of their fellow-creatures, and desire to see truth prevail over ignorance and barbarous policy.

The rains which fall in Andalusia during the autumnal months, are abundant, and the face of the country, from being dry and parched, quickly assumes the pleasing aspect of reviviscence. The months of May, June, July, August, and September, are particularly hot and dry. In October the rains descend in torrents for days and even weeks together. The thermometer averages, in the hot months beforementioned, from 73 to 82 of Fahrenheit;—from November till May, it ranges from 50 to 75. The Levant winds produce great languor and lassitude, even in the strongest constitutions; and it has been remarked by the inhabitants of sea-port towns in the South of Spain, that the yellow fever, or some epidemic has, of late years, invariably prevailed when excessive heat succeeded a prevalence of the Sirocco winds.

The second chapter of the work before us consists entirely of meteorological tables, and therefore must be passed over. The third chapter contains many judicious and curious observations on contagion, &c. to which we cannot do justice. We fully agree with Mr. O'Halloran, in considering the fever as of local origin in the beginning; but we are not disposed to come to his conclusion, that it is never personally contagious afterwards, because such a conclusion does not tally with our own observations on other fevers, or the observations of our best writers, ancient and modern.

Our author informs us that, till very lately, the Spanish physicians were impressed with the firm belief, that yellow fever was contagious in the most unlimited sense of the word, but that latterly their faith in contagion has been shaken. Some of them have even ventured to doubt, in their writings, the probability that yellow fever can be communicated by intercourse between the healthy and the sick—*in atmospheres not epidemical.* Still, however, they think and act with all their original prepossessions. In respect to the opinion of our author himself, it is as follows :—

> " If it were possible in one hour to remove an hospital, filled with patients labouring under the worst forms of this disease, from *Seville* to *London*, not a single individual of these countries would be affected by the importation." 36.

We believe this to be true, or nearly so—that is, we think that did the disease spread to a few in immediate attendance on the sick, yet that it would go no farther, and would very soon become extinct. However this may be, our author, who certainly had better means of information than any of his countrymen, thinks he can shew, by cases which occurred under his own eye, that the fever in question is not a contagious disease, even on its own soil—in other words, that it cannot be communicated by *clothes*, *contact*, or *near approach.* That persons engaged in the performance of offices for the sick, suffer in a greater proportiou than those who are not, our author admits, but he endeavours to shew, that the fever is incommunicable by contact, or near approach, " unless a previous derangement of health predisposes to such an event." Innumerable instances, he observes, occurred in the course of his attendance upon patients labouring under yellow fever, " wherein persons *in full health*, were constantly occupied about the sick, without being at all affected"—a fact which, he thinks conclusive evidence, that fever is not necessarily or inevitably contagious. We do not entirely subscribe to this inference. People in prime health, and of firm, intrepid minds, are known to resist all contagious diseases, much more than the weakly and pusillanimous.

It was on the 23d September, 1820, that our author arrived at Xeres, and undertook the treatment of a disease, upon which the Spanish faculty seemed able to make but little impression. He commenced this duty without the slightest apprehension of personal danger, on his part, in consequence of having observed, and found it generally observed, that the yellow fever of tropical climates was not contagious. A short sojourn in Xeres convinced our author that truth was not to be found in hearsay, or candour to be expected from men, whose minds were made up and locked against conviction. He therefore determined upon collecting such materials himself, as would enable him to form a correct judgment on the subject. Mr. O'Halloran's *modification* of personal contagion will be understood, from the following short extract.

"But although propagation from person to person is not evident, there are certainly grounds for the supposition, that in the ill-ventilated apartments of the sick, a noxious material emanates from the subject, which alters the salubrious quality of the atmosphere to such a degree, as sometimes to render it unfit for supporting the necessary actions conducive to health, and thereby indirectly to produce fever." P. 45.

It is proper to observe, that this nice distinction can hardly be expected in practice; but, at all events, it is to be remembered, that the disease seldom originates in this way (personal infection, or febrific atmosphere) in airy apartments. Its attacks are confined, as he justly remarks, to ill-ventilated houses, where the noxious effluvia produced by disease, are increased in activity by the aggregation of subjects in a narrow space. It is equally true, that a person exposed to a limited quantum of air, enveloping those ill of fever, soon experiences, especially if of a susceptible habit, a degree of indisposition; and, even if he resists a regular attack of fever, his stomach and bowels become more or less deranged, partly from the offensive air which he breathes, and partly, from the impression made upon his mind by the melancholy scenes around him.

"It is the effect of this vitiated air of ill-ventilated houses which has impressed the community with the notion of the highly contagious properties of yellow fever; for it is taken for granted, that all those who sicken hold direct communication with the infected, never considering that thousands, such as nurses and other attendants, in whose arms the dissolution of patients often occurs, and upon whose bodies, clothes, &c. the blood and black vomit are frequently thrown, escape its attacks. The instances which I myself have seen, during my treatment of the disease at Xerez, in proof of the fact as to nurses and attendants not apparently suffering in greater proportion than those who were simply exposed to the ordinary atmosphere or the air of the sick chamber, are so numerons as to leave little doubt on my mind as to the non-existence of personal contagion in the epidemic yellow fever of Andalusia, as it appeared in 1820." 46.

Our author observed, that although persons who breathed the infected air of sickly houses, suffered more than those who walked the streets—yet, that the *latter* did not suffer in greater proportion, than those who secluded themselves from all intercourse, and who, consequently, were only exposed to the common epidemic cause which obviously pervaded all points within the epidemic town.

Upon entering the habitations of the poorer classes, the impure condition of the atmosphere (even when imperceptible by the sense of smelling) was indicated by a sensation of dryness in the mouth, an inclination to spit, a preternatural excitement or working of the abdominal muscles, and not unfrequently nausea or vomiting, succeeded by black viscous stools and slight pyrexia.

In Xeres the first manifestations of disease were usually in the stomach and alimentary canal, producing, in many cases, a total change in their secretions and excretions, often terminating in fever, if neglected. Mr. O'Halloran here notices a curious fact, which was, that in apartments which could not have undergone any change by the admission of fresh air, the atmospheric condition evidently underwent alterations, for which our author never could account—those changes occurring in the course of a single day.

" For it often happened, that the sick person wbo, at one moment, could not be approached, from an effluvium of which words can convey no idea, might be particularly examined at the termination of an hour, without any offence to the olfactory nerves of his attendants. It appeared to me to be difficult precisely to discover at what periods of disease these changes occurred: they were not commonly perceptible during the stages of depression; they seemed to have possessed a higher degree of intensity during the stages of excitement, on the first and second day, than at any other period." P. 49.

The Spanish physicians, although apparently advocates for ventilation, do not practise what they preach, while the people consider seclusion of light and air as actually necessary for the recovery of the sick.

" They imagine that the indication of cure, in fever of all types, consists in producing a determination to the surface; and, to ensure this effect, the windows and doors are closed, the chamber is heated by means of lighted charcoal which emits little smoke, and the patient is covered up in bed with an enormous quantity of clothes in order to force a sweat. It is under circumstances of this nature, where persons are crowded together in dark, ill-ventilated houses, that the disease spreads rapidly; and this has impressed the bulk of the people with an idea of the highly contagious properties of yellow fever." 50.

Fear is well-known to predispose to fever, and this is the reason assigned by the Spaniards why persons who seclude themselves from intercourse, fall in as great a proportion as those who walk the streets. Our author agrees with most other practitioners in considering people who have once undergone an attack of yellow fever, to be little susceptible of a repetition of the same. But this immunity is far from being without many exceptions. Mr. O'H. relates some curious instances of the effects of fear in developing fever—one is not a little remarkable. Mr. Gomez avoided intercourse as much as possible, under the idea of securing himself from infection; but was one day forcibly seized by a gentleman much in the habit of frequenting sick chambers, with the view of frightening Mr. Gomez. The latter gentleman, however, quickly sickened of fever and died! The remainder of this chapter is occupied in the recital of numerous cases of the fever, for which we must refer to the volume itself. The fourth chapter is on the symptomatology of the disease as it appears in the sanguine temperament. We shall not enter on this chapter, because the subject of it was sufficiently treated of in our last number, when reviewing Dr. Jackson's work. The fifth chapter is on the treatment of this form of the fever, from which we shall make some notes.

In this form there is little time for consideration; for if the first few hours are allowed to pass away without some bold and decisive measure, all subsequent attempts will be fruitless. This form, from its history and appearances on dissection, is incontestibly inflammatory. When seen early Mr. O'Halloran recommends the patient to be immediately immersed in a warm bath, of the temperature of 100°, where he is to remain for fifteen or twenty minutes, till the whole body is cleansed by means of coarse cloths or flesh-brushes. When put to bed a vein is to be opened in both arms, and the blood allowed to flow until the pulse undergoes a decided change, or syncope supervenes. Our author has tried it in several instances, with decided benefit. In some it appeared to cut short the disease—in others, it was succeeded by copious sweats and alvine discharges—in all, if carried to a sufficient extent, it removed the head-ache and general distress. In short, Mr. O'Halloran thinks he is authorized in believing that, were other auxiliaries at hand which are necessary for the successful treatment of fever, the extensive use of venesection would render the mortality of the Spanish fever comparatively trifling—not perhaps exceeding one in twenty, at least as far as could be judged by the epidemic of 1820.

The Spanish physicians have, for many years past, been greatly prejudiced against bleeding in fevers. But the arrival of Dr. Jackson in Cadiz, and the great success attending copious venesection in the case of Dr. M'Gibbon, induced some of them to try the effect of the remedy, and an official publication in the mercantile diary of Cadiz, dated the 25th September, 1820, gives ample proof of the success of the experiment.

After venesection a brisk cathartic of calomel and jalap is recommended to be given. If these are slow in operation, enemata are to be thrown up—and, these failing, a stimulating draught composed of tincture of myrrh and aloes, or of jalap and rhubarb. Being bled and purged, if much irritability of the stomach be present, a very large blister should be applied to the epigastric region, and effervescing draughts taken every hour according to the urgency of the symptoms. If, after this, the head-aches should return, which does not often happen, the head is to be shaved, and blisters applied to the temples and nape of the neck, while cold lotions are to be kept constantly applied to the scalp. Ten hours from the commencement of the attack *pediluvia* are preferred by our author to the general bath. This foot bath was found very serviceable in Xeres, by producing copious sweats, allaying irritation, and inducing sleep. If this plan be vigorously pursued during the first day, it will generally be found sufficient to arrest the disease *in toto*.

"On the second day, and even on the first, if the disorder be violent, CALOMEL and JAMES'S POWDER, in form of pill, given in the proportion of five grains of the former to four of the latter, every second hour, will be found an *invaluable medicine*. This combination, notwithstanding the nauseating effects of the James's powder, is frequently found to remain on the stomach when nothing else will. It maintains an open state of the bowels, without producing that distressing and alarming purging which too often arises from the operation of drastic purgatives; while, by its operation on the system at large, it produces the most salutary effects." 93.

The warm bath is to be repeated on the second day, and if the symptoms indicate the necessity of the lancet, a vein is to be opened, and while the body is immersed, a large quantity is to be abstracted.

"If the irritation of the stomach still continues, the effervescing draughts, with the liq. ammon. acet. and tincture of opium, may be repeated; or a weak solution of the superacetas plumbi, or the sulphas zinci, should be administered, as it frequently restrains the vomiting without superseding the purgative effects of the calomel." P. 94.

On the third day, if the system is not under the influence of mercury, Mr. O'Halloran thinks it adviseable to continue the calomel pills, conjoining ammonia, camphor, and opium, with the view of exciting artificial action. In the majority of instances, however, Mr. O'H. observes, the patients treated in the manner described will be under the influence of mercury on the third day, "after which his recovery is certain and rapid. He will generally walk about on the seventh day." 95. Our author states that, without a single exception, throughout the whole period of the epidemic, every patient in whom *ptyalism* could be induced, recovered. He insists upon the state of ptyalism, because he has seen many instances, in which the mouth and gums became sore and ulcerated, but *without any salivary discharge*, and where the patients did not recover. Several cases carefully detailed close this chapter of the work.

The sixth chapter is on the symptoms of the epidemic, as manifested in the *nervous* or *serous* temperament. This form of the fever is by far the most common and dangerous. It is particularly characterized, in the commencement, by a deficiency of vital heat—impaired sensibility—and a withering, as it were, of the whole frame. The patient, apparently in perfect health, is struck by some power that at once deprives him of all his energies, "transmutes him to a living corpse, and marks him for the *grave*." The patient, when questioned as to his state, can give no rational account of himself, but mutters incoherently when spoken to. He is usually peevish, fretful, and chilly, about the period of investigation—the chills sometimes continuing for a few minutes only—but generally terminating about the third or fifth hour.

There is no evident symptom of fever, Mr. O'H. observes, present at this period.* The tongue is clean, the skin cool and dry; but the eyes are blood-shot, and the countenance is stupid and comatose. Head-ache and pains in the extremities soon set in, increasing in violence as the chills diminish. A caustic febrile heat supervenes, first about the head, and finally diffusing itself all over the trunk. With the head-ache the intellects are disturbed—and the face, excepting a circumscribed deep red spot on the cheek, is always deadly pale. The eye is frequently red and painful; impatient of light, or watery and glistening—sometimes nearly natural—sometimes white, glossy, and painful.

"In some cases the conjunctiva assumes a bluish cast, and is surcharged with blue vessels, while the cornea is watery and glistening. This eye is frightful to look at, and invariably indicates a malignant disease. The eye-brows are frequently knit or contracted. The lips are generally pale, sometimes the reverse. The mouth is dry

---

* This is a mistake. The stage of *depression* is just as much a state of fever as the violent *reaction* or excitement which succeeds it. The author should have used "*excitement*" for fever.

and parched, the breath hot and pungent, resembling a diffused blast from a distant furnace; or clammy, moist, and cool. The tongue is clean for the first six or seven hours, often dry, but it becomes white in general after that period. It is so changed in appearance in some instances, as to induce a person who had not seen the like before, to believe that it had been rubbed over with lunar caustic." P. 125.

There is little thirst in the beginning, or even in the advanced stages, especially in the fatal cases. The skin, in general, is below the natural temperature, dry, and constricted. The feet are frequently cold—the pulse slow and contracted, sometimes irregular—generally averaging from 80 to 90. The stomach is usually irritable—the matter ejected, at first, being of a turbid, watery, or ropy nature—afterwards dark, turbid, and black. Vomiting, although a common symptom, is not always present, since cases sometimes terminate fatally without it; but nausea is rarely absent. The patient often complains of weight about the stomach, to which the Spaniards give the name of *fatigas*. The abdomen is tense and painful, especially on pressure; the patient passes black stools which deposit a black and gritty sediment—but are rarely, if ever, feculent, being sometimes watery and copious, sometimes slimy and small, frequently mixed with flaky substauces, apparently abrasions from the internal coats of the intestines. The urinary secretion is very irregular, sometimes scanty and high-coloured, often suspended altogether. As the disease advances the pulse frequently disappears at the wrist—the coffee-coloured vomiting then commences—sometimes petechiæ cover the body. The parotid glands enlarge in a few instances—and all these symptoms continuing and increasing, life is destroyed on the fourth or fifth day, sometimes later. No periodical changes were observed during the two first days; but on the third, fifth, or seventh, either febrile heat supervened, or depression took place, speedily followed by death.

It will be evident to most of our readers that the above is a very spirited sketch of the *congestive* fevers long before described by Jackson, and more recently by Dr. Armstrong, in his immortal work on typhus. It would appear that the *cause* of the fever, whether specific contagion, the effluvia from human bodies, or a tainted atmosphere, overwhelms the system, nervous and sanguiferous, and prevents that salutary reaction or free febrile excitement, which is evidently the means by which Nature resists and expels the original morbid impression. The late epidemic cholera of India appears to have a very great affinity to the congestive form of fever described above; the cholera, however, being more violent and fatal. In the latter disease the *vomiting* appears to be the only effort which Nature is able to make in struggling with the morbid cause. The great and leading indications then in fever, have long appeared to us much more simple than they are represented in books or in conversations. In these stages of depression, the first effects of the febrific cause, is it not consonant with the best practice to take those measures which *promote* reaction or excitement—especially the warm bath, and even diffusible stimuli, carefully and cautiously applied? In these stages, however, there are certain counter-indications. While the heart is incapable of distributing the blood to the surface, from real weakness or oppression, we have the lungs, the brain, and other important organs drowned, if we may be allowed such an expression, with congested blood. Here then, while with one hand we stimulate the heart and cutaneous vessels by cordials and the warm bath, we are justified in detracting blood, with the other hand, in order to relieve those organs which are oppressed with an undue proportion of the vital fluid. In respect to the stage of re-action, the clear indication is to control it when it runs high, to *prevent local* mischief in some important organ; and when topical inflammation is manifested, to combine local with general bleeding till the organ is relieved, whatever the quantity may be that is required for that purpose. It is in vain to talk of husbanding the resources of the constitution, when an organ is a prey to congestion or inflammation. Certainly the greatest assistance we can give the constitution is relief to any of its important viscera when suffering from disease.

When the *nervous* or *serous* temperament was the subject of fever, our author was far from successful, especially at first. But it was still worse with the Spanish physicians, our author asserting that out of more than 100 patients who were severely afflicted in the hospitals of Cadiz and Xeres, and which our author had an opportunity of seeing, not one recovered. Finding the usual remedies of bleeding, purgatives, frictions, fomentations, antimonials, and blisters unavailing, Mr. O'Halloran had recourse to *emetics*, and to these he was principally indebted for the recovery of the few who did not fall victims to the epidemic. We must conclude this article, already too far extended, with the following extract:—

"With regard to the general mode of treatment, I would recommend that the patient, on the first day of the attack, should be immediately placed in a hot bath, and left there until the body is thoroughly heated, or in other words, until a degree of artificial fever is produced. He should then be rubbed dry, and covered, in bed, with more than the usual quantity of clothes. If, after he has been in bed for half an hour, reaction takes place, I would then advise the abstraction of a small quantity of blood: but if he continues cold, I would only repeat the bath. Recourse should next be had to emetics; and as the *subsulphas hydrargyri*, or turbith mineral, and ipecacuanha (in the proportion of six grains of the former to five of the latter,) will be found effectually to unload the stomach, without much straining, I would recommend it in preference to any other. After the operation of the emetic, calomel and James's powder should be exhibited in the proportions of five grains of the former to three of the latter, in the form of pills, every two hours; with a view of preserving an open state of the skin and bowels, and of affecting the system. The head should be shaved, and blisters applied to the temples and nape of the neck, while the head is kept cool by means of cold applications. The skin should be rubbed over with heated oils when there is much dryness and constriction. The *pediluvium* and fomentations may always be used with advantage. *Opium*, *camphor*, and *ammonia* should be had recourse to, where the prostration of strength is great. The *liquor ammoniæ acetatis*, effervescing draughts, wine, and the mineral acids, may also be tried, according to circumstances; but *emetics* are the remedies upon which our greatest reliance is to be placed. In two forlorn cases I tried a weak solution of the *argentum nitratum* with a view of checking severe hickup, and it certainly answered the purpose for which it was intended: the patients did not recover; but as it stopped the hickup and procured rest, I think it right to mention it as a remedy which may be tried without detriment in such cases." 134.

The work concludes with two appendices on the supposed introduction of yellow fever into the town of Xeres, in the year 1820, which we recommend to the perusal of the importers.

Mr. O'Halloran, with that zeal and courage characteristic of youthful and ardent minds, but which we have seen also in our venerable countryman, Dr. Jackson, has again repaired to the scene of this dreadful scourge, with the hope of elucidating its nature and causes, and ascertaining the best principles of combating its ravages. The labours of our countrymen, who have thus risked their lives and endangered their health, in the service of humanity, should meet with liberal encouragement from a British public, and we trust and hope that every medical society, at least, and many opulent individuals, will consider it a duty owing to science and philanthropy, to place the little works of Drs. Jackson and O'Halloran in their libraries. We think we need hardly add, by way of inducement, that these publications were at the private expence of the authors themselves; and it would be most unjustly cruel in the profession at large, to allow these individuals to suffer in their purses as well as in their persons, while supplying us with information which we otherwise could not possibly obtain.

The perusal of Mr. O'Halloran's work has convinced us that the character drawn of this gentleman by his colleague in the "holy office" of visiting the sick at Cadiz and Xeres, (Dr. Jackson,) was not overcharged. May he long live to enjoy that pleasure which "nothing earthly gives or can destroy," the approbation of his own conscience; and may he persevere in the cause of medical science till he is crowned with success, by seeing it far advanced beyond the point at which it now stands.

